# Studies of Inhibitory Mechanisms of Propeptide-Like Cysteine Protease Inhibitors

**DOI:** 10.1155/2014/848937

**Published:** 2014-06-19

**Authors:** Bui T. T. Nga, Yuki Takeshita, Misa Yamamoto, Yoshimi Yamamoto

**Affiliations:** ^1^Laboratory of Biochemistry, Joint Faculty of Veterinary Science, Yamaguchi University, Yamaguchi 753-8515, Japan; ^2^Faculty of Health Science, Yamaguchi University School of Medicine, Yamaguchi 755-8554, Japan

## Abstract

Mouse cytotoxic T-lymphocyte antigen-2*α* (CTLA-2*α*), *Drosophila* CTLA-2-like protein (crammer), and *Bombyx* cysteine protease inhibitor (BCPI) belong to a novel family of cysteine protease inhibitors (I29). Their inhibitory mechanisms were studied comparatively. CTLA-2*α* contains a cysteine residue (C75), which is essential for its inhibitory potency. The CTLA-2*α* monomer was converted to a disulfide-bonded dimer *in vitro* and *in vivo*. The dimer was fully inhibitory, but the monomer, which possessed a free thiol residue, was not. A disulfide-bonded CTLA-2*α*/cathepsin L complex was isolated, and a cathepsin L subunit with a molecular weight of 24,000 was identified as the interactive enzyme protein. Crammer also contains a cysteine residue (C72). Both dimeric and monomeric forms of crammer were inhibitory. A crammer mutant with Cys72 to alanine (C72A) was fully inhibitory, while the replacement of Gly73 with alanine (G73A) caused a significant loss in inhibitory potency, which suggests a different inhibition mechanism from CTLA-2*α*. BCPI does not contain cysteine residue. C-terminal region (L77-R80) of BCPI was essential for its inhibitory potency. CTLA-2*α* was inhibitory in the acidic pH condition but stabilized cathepsin L under neutral pH conditions. The different inhibition mechanisms and functional considerations of these inhibitors are discussed.

## 1. Introduction

**Figure 1:**
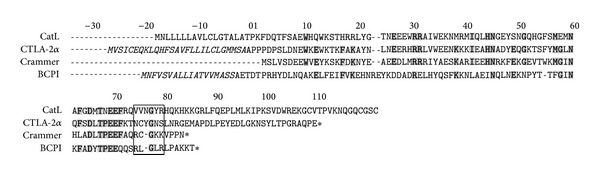
Propeptide-like cysteine protease inhibitors. Highly conserved amino acid residues are shown in bold. Gaps introduced to optimize the alignment are marked with dashes. N-terminal numberings are based on mature CTLA-2*α*. Putative signal sequences are shown in italics. GenBank accession numbers: CtsL (proregion of mouse procathepsin L), NP034114; CTLA-2*α*, S04924; Crammer, AAF57567; BCPI (BCPI*β*), CAB41937.

Denizot et al. were the first to show that mouse-activated T cells and mast cells expressed the mRNA encoding proteins, cytotoxic T-lymphocyte antigen-2s (CTLA-2*α* and CTLA-2*β*), which were shown to be highly homologous to the proregions of mouse cathepsin L [1] (Figure 1). We also identified a cysteine protease inhibitor protein,* Bombyx* cysteine protease inhibitor (BCPI), which was homologous to the proregions of cysteine proteases [2–4]. Homologous proteins have been reported in* Drosophila*, the* Drosophila* CTLA-2-like protein, crammer [5–7], and in Atlantic salmons (*Salmo salar*), salarin [8, 9]. In the past two decades, these proteins have been identified as cysteine protease inhibitors [4, 7, 10, 11]. As the amino acid sequences of the inhibitor proteins were shown to be homologous to the proregions of cysteine proteases, these inhibitors have been classified as propeptide-like cysteine protease inhibitors [5]. Comprehensive details are available in the MEROPS peptidase database under Family I29 (http://merops.sanger.ac.uk). Recombinant CTLA-2s have been shown to be potent, competitive inhibitors of cathepsin L-like cysteine proteases [10, 11]. A previous study showed that CTLA-2*α* was highly expressed in the pregnant uteruses of mice [12], and CTLA-2*β* expressed in early pregnant uteruses was proposed to be a regulator of embryo implantation [13]. Very interestingly,* Drosophila* crammer might have a role in long-term memory formation [6]. We identified the expression pattern of CTLA-2*α* mRNA in the mouse brain, and demonstrated its preferential enrichment in various neuronal populations [14]. We also demonstrated that the protein was mainly localized in the dendritic and axonal components of neurons [15]. Interestingly, a previous study showed that CTLA-2*α* was involved in the formation of regional immunity in the eye [16]. Retinal pigment epithelium-derived CTLA-2*α* has the capacity to generate T reg, which inhibit cathepsin L in T cells [17, 18]. Recently, CTLA-2*α* was shown to induce the cAMP/PKA-promoted apoptosis of murine T-lymphoma cells and cardiac fibroblasts [19]. These findings suggested that these propeptide-like cysteine protease inhibitors were involved in various intra- and extracellular functions. We previously identified the essential amino acid residues of CTLA-2*α* necessary for its inhibitory potency [20]. Three Trp residues (W12, W15, and W35) in the *α*1/*α*2-helixes, which form the hydrophobic core structure between the first and second *α*-helix, significantly contributed to inhibition. Another essential amino acid residue was shown to be cysteine 75 (C75), which is located in the immediate vicinity of the CTLA-2*α* region, and is interactive with the active-site cleft of the enzyme. We also suggested the possibility of disulfide bonding between CTLA-2*α* and the enzyme.

In the present study, we attempted to elucidate the functional roles of the cysteine residue in more detail. Studies with other inhibitors, crammer and BCPI, were also performed. For the inhibition studies, mouse cathepsin L (CtsL) and* Bombyx* cysteine protease (BCP), both of which belong to a papain family (C1A) in the MEROPS peptidase database, were employed. CTLA-2*α* may inhibit cathepsin L-like cysteine protease by oxidizing the active thiol residue of the enzyme with its own thiol residue. Different inhibition mechanism was proposed for crammer. Another function of CTLA-2*α* as stabilizer, different from inhibitor, was also presented.

## 2. Materials and Methods

### 2.1. Chemicals

Benzyloxycarbonyl-L-phenylalanyl-L-arginine 4-methylcoumarinyl-7-amide (Z-Phe-Arg-MCA) and N-[N-(L-3-*trans*-carboxyoxiran-2-carbonyl)-L-leucyl]-Agmatine (E-64) were purchased from Peptide Institute Inc., Osaka, Japan, DMEM medium, GSSG, DTT (Wako, Japan), Lipofectamine LTX and PLUS (Invitrogen, Oslo, Norway), Quickchange II site-directed mutagenesis kit (Agilent Technologies, CA, USA), Primestar mutagenesis kit (Takara, Japan), plasmid pFUSE-hlgG2-Fc1 (InvivoGen, CA), Toyopearl HW-50 (Tosoh Co., Tokyo, Japan), PD MiniTrap G-25 column (GE Healthcare, UK), and Dynabeads TALON (Invitrogen, Oslo, Norway).

### 2.2. Inhibitor Proteins

Recombinant BCPI [4], recombinant CTLA-2*α* [11], and recombinant crammer [7] were purified according to the methods described previously. For further purification of CTLA-2*α* and crammer to separate the dimeric form from the monomeric form, the preparation from His-bind affinity chromatography was subjected to gel filtration using Toyopearl HW-50 [2]. The production of mutant CTLA-2*α*s was achieved by the method described previously [20]. The mutant cDNAs of BCPI and crammer were constructed using the Quickchange II site-directed mutagenesis kit and Primestar mutagenesis kit. The expression and purification of the mutant proteins were carried out according to the methods applied to wild inhibitor proteins. All of these inhibitor proteins were fusion proteins with extra amino terminal 26 amino acid residues including a His-tag sequence [11]. The protein concentrations of the inhibitor preparations were determined using the predicted molar extinction coefficient at 280 nm (calculated from the amino acid sequences) [21] and the Bradford method with BSA as a standard [22].

### 2.3. Assay of Inhibitory Activities

For inhibition studies of CTLA-2*α*, purification of recombinant mouse cathepsin L (CtsL) was performed according to the method described by Kramer et al. [23]. The concentration of the active enzyme was determined by active-site titration using E-64 [24]. The activity of CtsL was determined by the method of Barrett and Kirschke with a brief modification [25]. A total of 10 *μ*L of CtsL was preincubated for 5 min at 37°C in the assay buffer, and 10 *μ*L of CTLA-2*α* was added. The assay buffer (500 *μ*L) consisted of 1 mM EDTA, 8 mM cysteine, and 0.1 M sodium acetate, pH 5.5. The enzyme reaction was started by the addition of 5 *μ*L of Z-Phe-Arg-MCA (1 mM). After 5 min at 37°C, the reaction was stopped and free MCA was measured. For inhibition studies of crammer and BCPI,* Bombyx* cysteine protease (BCP) was employed. BCP was purified as described previously [26]. The concentration of the active enzyme was determined as described above. The inhibition kinetics of BCPI [4], CTLA-2*α* [11], and crammer [7] were studied according to the methods described previously.

### 2.4. Expression of CTLA-2*α* in HEK293 Cells

Full-length cDNA encoding CTLA-2*α* was cloned into a pFUΔss plasmid (modified from pFUSE-hlgG2-Fc1). HEK293 cells (human embryonic kidney cells) were cultured and maintained in DMEM medium supplemented with 10% fetal calf serum. Transfection was performed using Lipofectamine LTX and PLUS according to the instructions provided by the manufacturer. After 48 h culturing, the whole cell lysate and culture medium were collected and subjected to SDS-PAGE. Western blotting was carried out according to the method described previously [15].

### 2.5. Dimer-Monomer Conversion of CTLA-2*α*

The dimeric form of CTLA-2*α* (150 *μ*g) was incubated for 15 min at 37°C in 500 *μ*L of the reducing buffer (150 mM NaCl, 1 mM EDTA, 50 mM DTT, and 100 mM Tris-Cl, pH 7.4). After incubation, the buffer was replaced by equilibration buffer (150 mM NaCl, 1 mM EDTA, and 100 mM Tris-Cl, pH 8.2) containing or not containing 2 mM GSSG using a PD MiniTrap G-25 column. The samples were further incubated at 4°C overnight. The inhibition assay and SDS-PAGE analysis were then performed. For the inhibition assays towards CtsL, 30 *μ*L of the CTLA-2*α* fraction was added to the assay buffer (500 *μ*L) as described above.

### 2.6. Solation of CTLA-2*α*/Cathepsin L Complex

CtsL and CTLA-2*α* were preincubated in 500 *μ*L of preincubation buffer (1 mM EDTA, 8 mM cysteine, and 100 mM sodium acetate, pH 5.0) at 37°C. After 5 min, the buffer was quickly exchanged with buffer containing 150 mM NaCl, 1 mM EDTA, and 100 mM Tris-Cl, pH 7.4 by gel filtration using a PD MiniTrap G-25 column. The sample was then further incubated for 20 min at 37°C. In order to isolate the CTLA-2*α*/cathepsin L complex, an aliquot of the incubated sample was applied to a His-bind resin (Dynabeads TALON). The unbound proteins were removed by washing and the target proteins containing CTLA-2*α*/cathepsin L complex were specifically eluted according to the manufacturer's instructions. For measurements of CtsL enzyme activity, an aliquot of the complex fraction was incubated in 0.1 M sodium acetate (pH 5.5) or 0.1 M sodium phosphate (pH 7.4) containing 1 mM EDTA, 8 mM cysteine. The enzyme reaction was started by the addition of Z-Phe-Arg-MCA (10 *μ*M). The progress curves were monitored continuously for 5 min at 37°C at excitation and emission wavelengths of 370 and 460 nm with a spectrofluorometer (model F2000, Hitachi). SDS-PAGE and immunoblotting analyses were performed as described previously [15]. Affinity purified rabbit anti- CTLA-2*α* and anti-cathepsin L antibodies were prepared as described previously [15].

### 2.7. pH Dependence of the Interaction between CTLA-2*α* and CtsL

CtsL was activated by preincubation in the buffer (1 mM EDTA, 8 mM cysteine, 0.1 M sodium acetate, pH 5.5) for 5 min at 37°C. The activated CtsL was mixed in the assay buffer (500 *μ*L) containing 1 mM EDTA, 8 mM cysteine and CTLA-2*α* (50, 100 nM) at different pHs. Both 0.1 M sodium acetate buffer (between pH 3.8 and pH 5.6) and 0.1 M sodium phosphate buffer (between pH 5.6 and 7.6) were used. The enzyme reaction was started by the addition of 5 *μ*L of Z-Phe-Arg-MCA (1 mM). After 5 min at 37°C, the reaction was stopped and free MCA was measured.

## 3. Results

### 3.1. Purification of Inhibitor Proteins and Expression of CTLA-2*α*

**Figure 2:**
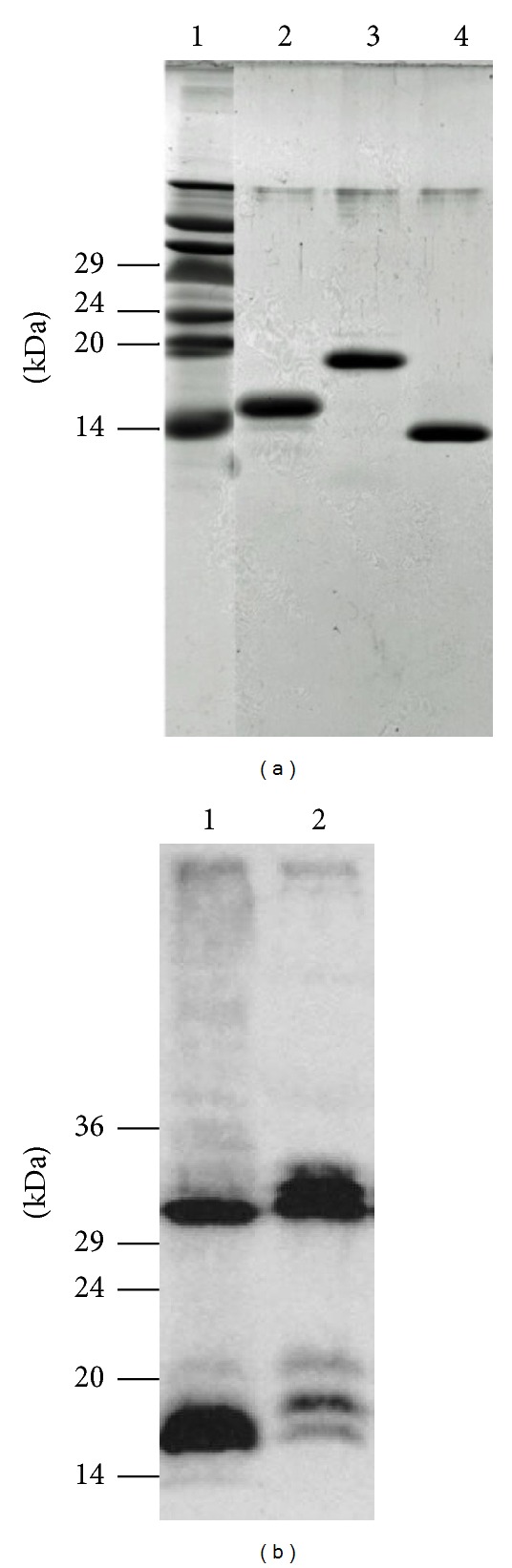
(a) Recombinant CTLA-2*α*, BCPI, and crammer. Inhibitor proteins were subjected to Tricine/SDS-PAGE using 16.5% polyacrylamide gels. Proteins were stained with Coomassie brilliant blue. Lane 1, protein molecular weight standards; lane 2, BCPI (3 *μ*g); lane 3, CTLA-2*α* (3 *μ*g); lane 4, crammer (3 *μ*g). (b) CTLA-2*α* expressed in HEK293 cells. Samples were subjected to SDS-PAGE using 12% polyacrylamide gels in the absence of 2-mercaptoethanol and analyzed by Western blotting using an anti-CTLA-2*α* antibody. Lane 1, cell extract; lane 2, medium.

The recombinant inhibitor proteins used in this experiment, CTLA-2*α*, crammer, and BCPI, can be seen in Figure 2(a), as homogeneous forms in the Tricine/SDS-PAGE gel [27]. The CTLA-2*α* protein was overexpressed in HEK293 cells, which demonstrated the existence of both intra- and extracellular functions (Figure 2(b)). The expressed protein could be present as the monomeric and dimeric form. Most of the CTLA-2*α* secreted extracellularly was identified as the dimeric form. Recombinant CTLA-2*α* and crammer expressed in* E. coli* were purified as the monomeric and dimeric forms, but mainly as the dimeric form, whereas only the monomeric form of recombinant BCPI was obtained [4, 7, 11].

### 3.2. Monomer-Dimmer Conversion of CTLA-2*α* and Crammer

**Figure 3:**
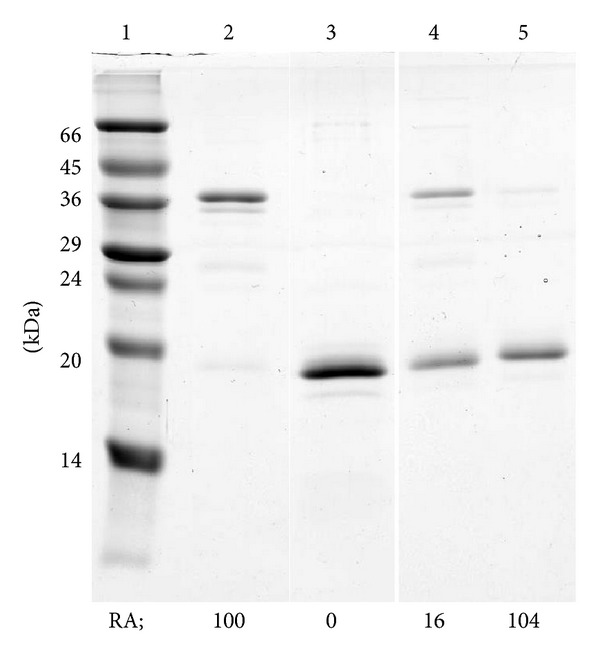
Dimer-monomer conversion of CTLA-2*α*. SDS-PAGE using 15% polyacrylamide gels was performed in the absence of 2-mercaptoethanol. Proteins were stained with Coomassie brilliant blue. Lane 1, protein molecular weight standards; lane 2, dimeric form of CTLA-2*α*; lane 3, sample of lane 2 treated with 50 mM DTT; lane 4, sample of lane 3 further incubated in the absence of DTT; lane 5, sample of lane 3 further incubated in the absence of DTT and in the presence of 2 mM GSSG. RA, relative inhibition activity towards CtsL, activity of the dimeric form of CTLA-2*α* as 100%.

**Figure 4:**
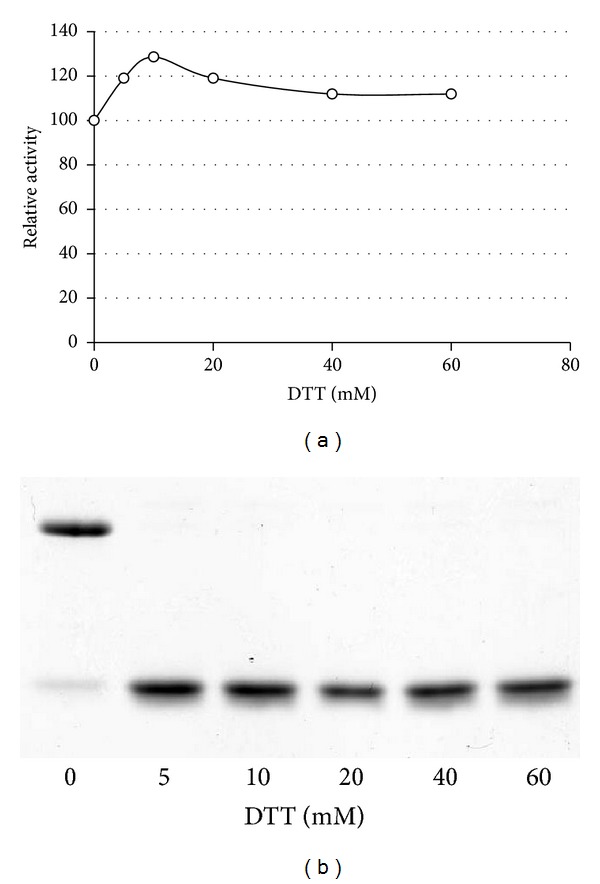
Dimer-monomer conversion of crammer. The dimeric form of crammer was incubated for 15 min at 37°C in the reducing buffer containing DTT. After incubation, aliquots were analyzed for inhibition activities towards BCP (a) and SDS-PAGE (b).

As described above, CTLA-2*α* can be present as either the monomeric or dimeric form. CTLA-2*α* has one cysteine residue (Cys75). In a previous study, we constructed mutant CTLA-2*α*s by replacing the cysteine residue with an alanine or serine residue (C75A, C75S). The dimeric form of CTLA-2*α* was no longer detected by SDS-PAGE of such mutant proteins, suggesting that the dimeric form of CTLA-2*α* resulted from the formation of an intermolecular disulfide bond between monomers. Interestingly, the inhibitory activity of the cysteine mutants (C75A, C75S) was markedly reduced. As a further investigation of the functional roles of the cysteine residue, we first isolated the dimeric form of CTLA-2*α* by separating it from the monomeric form (Figure 3, lane 2). It was fully inhibitory against CtsL. By treating it with the strong reducing reagent, DTT, the dimeric form lost all its inhibitory activity and was completely converted to the monomeric form (Figure 3, lane 3). The preparation was then subjected to gel filtration to remove DTT and further incubated in the absence or presence of L-glutathione (oxidized, GSSG). After overnight incubation, a part of the monomeric form was reassembled to the dimeric form and recovered trace amounts of inhibitory activity (Figure 3, lane 4). Interestingly, in the presence of GSSG, CTLA-2*α* fully recovered its inhibitory activity although its molecular form remained mostly as the monomeric form (Figure 3, lane 5). The possibility of inhibition of CtsL by GSSG could be omitted because GSSG (up to 1 mM) did not exhibit inhibitory activity towards CtsL in the present assay system (data not shown), and a final concentration of GSSG of the assay mixture containing CTLA-2*α* treated with GSSG was 0.12 mM. Different findings were observed for the* Drosophila* protein, crammer (Figure 4). The monomeric form of crammer resulted by treating its dimmer with DTT, and its inhibitory potency was fully active.

### 3.3. Isolation of CTLA-2*α*/Cathepsin L Complex

**Figure 5:**
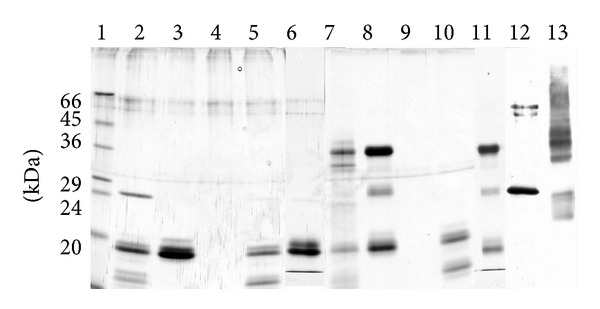
Isolation of the CTLA-2*α*/cathepsin L complex. SDS-PAGE using 12% polyacrylamide gels was performed with (lanes 1–6, 12) or without (lanes 7–11, 13) 2-mercaptoethanol. Proteins were stained with silver nitrate reagent (lanes 1–11). Proteins immunoreactive to the anti-cathepsin L antibody were visualized (lanes 12, 13). Lane 1, protein molecular weight standards; lanes 2, 7, 12, and 13, CTLA-2*α*/CtsL; lanes 3, 8, CTLA-2*α*; lanes 4, 9, CtsL: lanes 5, 10, CTLA-2*α* (C75S)/CtsL; lanes 6, 11, CTLA-2*α*/CtsL(E-64 treated).

**Figure 6:**
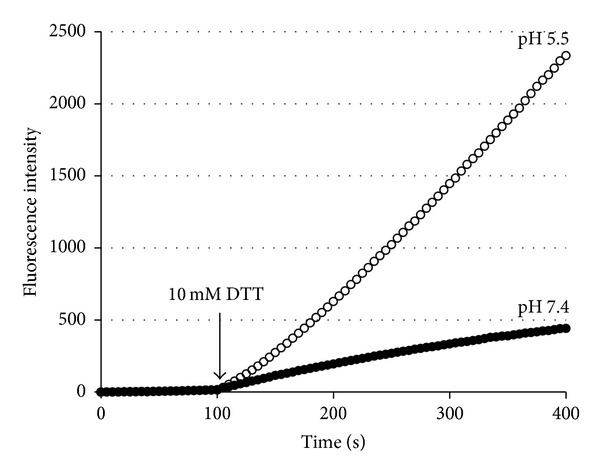
Progress curves for the cathepsin L activity of the CTLA-2*α*/cathepsin L complex. The progress curves were continuously monitored. The arrow indicates the time when 10 mM DTT was added to the reaction mixture.

Since cathepsin L is a cysteine protease having an essential cysteine residue at its active site, we next attempted to isolate the CTLA-2*α*/cathepsin L complex conjugated with a disulfide bond. CTLA-2*α* and/or CtsL were firstly preincubated for a short time in an acidic buffer (pH 5.0) containing cysteine to activate CtsL and allow the interaction between CTLA-2*α* and CtsL. The buffer was quickly exchanged with a neutral buffer (pH 7.4) not containing cysteine. After incubation, CTLA-2*α* and CTLA-2*α* conjugated proteins were recovered by precipitation with the His-bind resin from the incubation mixture.  Figure 5 shows SDS-PAGE of several combination experiments. The His-bind resin specifically recovered CTLA-2*α*, but not CtsL (Figure 5, lanes 3, 4, 8, and 9). When CTLA-2*α* was incubated with CtsL, several additional protein bands could be seen, indicating that these proteins were conjugated with CTLA-2*α* (lanes 2, 7). Western blot analysis using an anti-cathepsin L antibody clearly showed that among these proteins, the protein with a molecular weight of 24,000 was CtsL (lanes 2, 12). The lack of a band corresponding to CtsL in the incubation with the CTLA-2*α* mutant, C75S, suggested that CTLA-2*α* was conjugated with CtsL by disulfide bonding (lanes 5, 10). Furthermore, the lack of a similar band was also observed in the incubation of CTLA-2*α* with CtsL lacking an active site cysteine residue (lanes 6, 11). The CTLA-2*α*/cathepsin L complex behaved as a heterogeneous molecular form in SDS-PAGE without 2-mercaptoethanol (lanes 7, 13). To ascertain whether the CTLA-2*α*/cathepsin L complex retains its enzyme activity or not, the cathepsin L activity of the isolated complex was continuously measured (Figure 6). The complex did not exhibit enzyme activity even in the acidic condition (pH 5.5). After dissociation of the complex by treatment with DTT, significant and pH dependent cathepsin L activities were measured.

### 3.4. CTLA-2*α* Stabilizes Cathepsin L at Neutral pH

**Figure 7:**
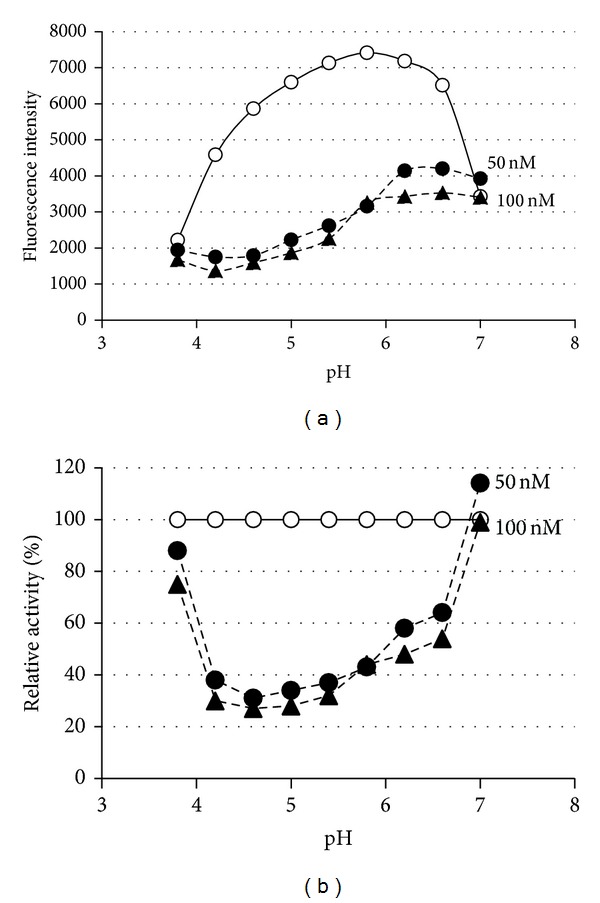
(a) pH dependence of the interaction between CTLA-2*α* and CtsL. Data are from one of two similar experiments. (b) Dependence of the residual activity (%) on different pHs in the presence of CTLA-2*α*. Open circles, control; closed circles, 50 nM CTLA-2*α*; closed triangles, 100 nM CTLA-2*α*.

**Figure 8:**
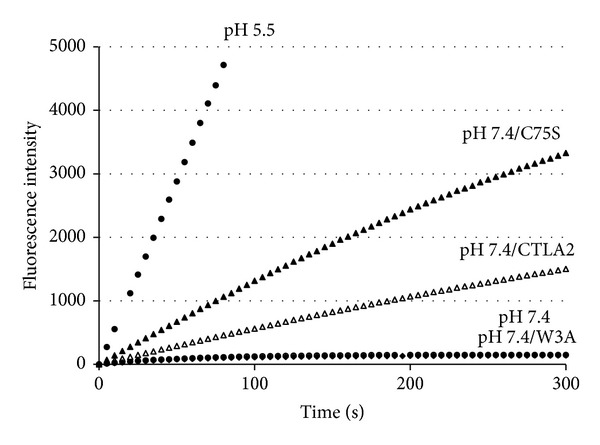
Stability of CtsL at pH 7.4 in the presence of CTLA-2*α*. Activated CtsL (80 pM) was incubated in 0.1 M sodium phosphate, pH 7.4 containing 1 mM EDTA, 8 mM cysteine, and 10 *μ*M Z-Phe-Arg-MCA, and progress curves were continuously monitored. For the assay at pH 5.5, 0.1 M sodium acetate, pH 5.5 was used. Open circles, at pH 7.4; closed circles, at pH 5.5; open triangles, at pH 7.4 in the presence of CTLA-2*α*; closed triangles, at pH 7.4 in the presence of CTLA-2*α*, C75S mutant; closed rhombus, pH 7.4 in the presence of CTLA-2*α*, W3A mutant.

Cathepsin L, being conjugated with CTLA-2*α* in the neutral condition, retained its activity, suggesting that CTLA-2*α* may stabilize cathepsin L at neutral pH. We first studied pH-dependency of the CTLA-2*α* inhibition (Figure 7). Similar to BCPI, the inhibition of CTLA-2*α* was not dependent on pH, in a range between pH 4.0 and pH 6.0 [4]. In more acidic pH (pH 3.8), CTLA-2*α* lost its inhibitory activity. Interestingly, full enzyme activity was retained at pH 7.0 at a CTLA-2*α* concentration of 100 nM, which indicates that CTLA-2*α* may not be inhibitory towards CtsL under neutral pH conditions. To investigate this in more detail, the process of the enzyme reaction was monitored in the presence or absence of CTLA-2*α* (Figure 8). CtsL was quickly inactivated at pH 7.4, whereas it was fully active at pH 5.5. After the 5 min incubation at pH 7.4, the enzyme lost almost all its activity. However, it retained its activity in the presence of CTLA-2*α*. Enzymatic activity was higher in the presence of the mutant CTLA-2*α*, C75S, whereas no significant effect on activity was observed in mutant W3A (W12A/W15A/W35A).* Drosophila* crammer was also observed to be effective, while BCPI was not (data not shown).

### 3.5. Alanine Scanning of Crammer and BCPI

The* Drosophila* CTLA-2-like protein, crammer, has a cysteine residue (C72) at the homologous region to CTLA-2*α*, whereas* Bombyx* BCPI does not (Figure 1). This region has also been suggested to be an interactive site with the active site cleft of the enzyme. Therefore, in order to evaluate the functional contribution of particular amino acid residue to inhibition, alanine scanning experiments were performed on these inhibitor proteins, with a focus on the region containing the cysteine residue or homologous region (Tables 1 and 2).* Bombyx* cathepsin L-like cysteine protease (BCP) was used for the inhibition assay. Contrary to the results observed in CTLA-2*α*, the crammer mutant, C72A, was fully inhibitory with a Ki value of 1.87 nM. On the other hand, the inhibitory potencies of mutants with the Gly73 substitution (G73A, C72A/G73A) were markedly reduced. When Gly73 was replaced with Ala, the Ki value (>1000 nM) was significantly higher than that of WT, more than 200-fold, indicating that the mutant lost almost all its inhibitory activity. The inhibitory activity of mutant C72A/G73A was negligible (no inhibition at 1000 nM). The inhibitory activity of mutant K74A/K75A was moderately decreased. The inhibitory activities of the other mutants replaced with two alanine residues (F68A, Q70A/R71A, V76A/P77A, and P78A/N79A) were similar to that of WT. By deletion experiments, it is known that the BCPI C-terminal region (L77-R80) is essential for its inhibitory potency [4] (Table 2). The replacement of amino acids with alanine in this region caused significant decreases in inhibitory activities. When Leu77/Gly78 was replaced by Alas, the Ki value (60 nM) was significantly higher than that of WT, more than 500-fold.

## 4. Discussion

**Figure 9:**
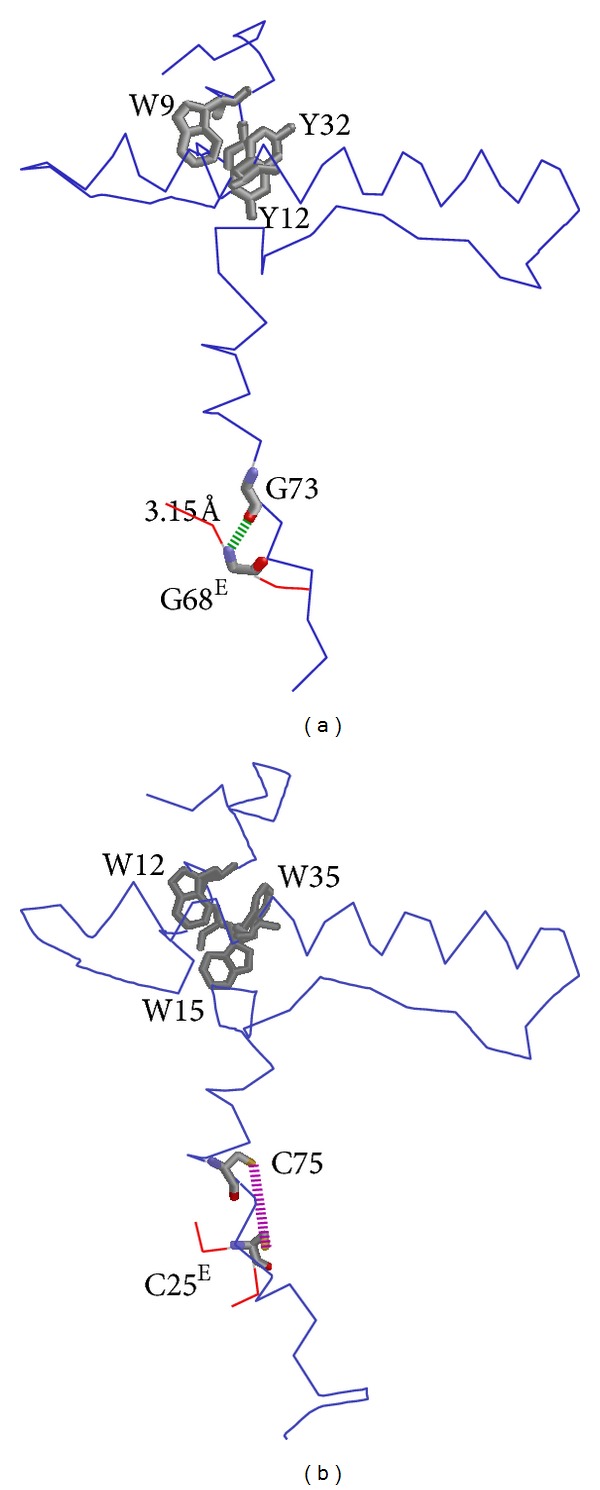
Highlights of the residues being involved in the interaction between polypeptides. (a) Complex of crammer and BCP; (b) complex of CTLA-2*α* and cathepsin L. Hydrophobic residues (Trp, Tyr) are shown with thick wireframes. Possible hydrogen bond (green) and disulfide bond (purple) are given with dashed lines. Molecular modeling was performed as described previously [20].

Even though CTLA-2 is the most studied of the propeptide-like cysteine protease inhibitors, its inhibition mechanisms have not been fully determined. Recombinant CTLA-2*α* is a potent, highly selective inhibitor of cathepsin L-like cysteine protease [11]. CTLA-2*β* also inhibits cathepsin L-like cysteine protease but is less selective than CTLA-2*α* [10]. CTLA-2*α* contains one cysteine residue, C75, in the sequence, rendering the formation of a dimer by an intermolecular disulfide bond between the monomers. In the present study, CTLA-2*α* was shown to be present as such a dimer* in vitro* and also possibly under physiological conditions. The dimeric form of CTLA-2*β* has also been reported, but this was a noncovalent complex of the monomer [10]. The dimeric form of CTLA-2*α*, converted to the monomeric form by treatment with DTT, completely lost its inhibitory activity. DTT is known to be a strong reducing reagent that reduces disulfides to dithiols and then maintains these thiols in a reduced state. Therefore, these results indicate that the CTLA-2*α* monomer with a free thiol residue was not inhibitory. By removing DTT from the buffer, part of the monomer reassociated to form a dimer, which suggests that the monomer and dimer forms of CTLA-2*α* exist in dynamic equilibrium in solution. Interestingly, the inhibitory potency of the CTLA-2*α* monomer recovered to the level of the dimer by oxidization with glutathione disulfide (GSSG). These results imply that the cysteine residue of CTLA-2*α* (C75), acting as a disulfide form, engaged in the inhibitory process. This result is in accordance with previous findings in which the cysteine residue (C75) was shown to be one of the essential amino acids of CTLA-2*α* for its inhibitory potency [20]. Moreover, present attempts to isolate the CTLA-2*α*/cathepsin L complex have revealed that, in the process of inhibition, CTLA-2*α* may be covalently bound to the catalytic subunit of cathepsin L via the cysteine residue (C75). The catalytic cysteine residue (C25^E^) of cathepsin L is located close to the cysteine residue of CTLA-2*α* (C75), as has been suggested by molecular modeling analysis of the CTLA-2*α*/cathepsin L complex (Figure 9(b)) [20]. CTLA-2*α* may inhibit cathepsin L-like cysteine protease by oxidizing the active thiol residue of the enzyme with its own thiol residue. We recently isolated the CTLA-2*α*/cathepsin L complex with a disulfide bonding from mouse pregnant uterus, suggesting this may occur* in vivo* (Nga et al., in preparation). It has been reported that cathepsin L is one of the targets of CTLA-2*β* in the pregnant uterus [13].

The* Drosophila* CTLA-2-like protein, crammer, also has a cysteine residue at the homologous sequence position to CTLA-2*α*. Recently, Tseng et al. reported that monomeric crammer was a strong inhibitor of cathepsin L, while the disulfide-bonded dimer was not [28]. Previous studies have also shown that the dimerization of cystatins led to losses in their inhibitory activities [29, 30]. In the present experiment, both the monomeric and dimeric forms of crammer were fully inhibitory [7]. More detailed consideration of this discrepancy may be beyond the scope of this study because a different crammer protein was prepared and a different enzyme was used for the inhibition assay. As shown in this study, the monomeric crammer prepared from the dimeric form by treatment with DTT did not lose any of its inhibitory activity. Kinetic experiments using mutant crammer, in which the cysteine residue was replaced by an alanine residue (C72A), revealed that the cysteine residue was not an essential amino acid for inhibitory potency. Studies have further confirmed the glycine residue of crammer (G73) to be one of the important amino acid residues for its inhibitory potency. In human procathepsin L, the sequence of the active site contacting region has been identified as M^75^-N-G-F-Q^79^ of the prosequence [31, 32]. The glycine residue (G77^P^), being closely located to the bottom of the active site cleft, may form a hydrogen bond with the glycine residue of the enzyme (G68^E^), which is known to be an interactive residue with substrates and inhibitors. As the three-dimensional structure of crammer was shown to be very similar to that of the proregion of procathepsin L [28], a similar explanation may be provided for the role of the glycine residue of crammer (Figure 9(a)). Molecular modeling analyses may support this explanation. The length of hydrogen bond became longer (6.16 Å) in the mutant, in which the glycine was replaced with alanine (G73A) (data not shown). However, although the homologous glycine residue was conserved in CTLA-2*α* (Figure 1), the mutant, in which this glycine was replaced with alanine (G77A), was fully inhibitory [20]. All of these results indicate that crammer inhibits cathepsin L-like cysteine protease by a different mechanism from that of CTLA-2*α*. Previous studies show that the C-terminal end of BCPI might interact with the BCP active site, and the sequence L^77^-G-L-R^80^was an important region for inhibitory potency [4, 5]. The results from the present alanine scanning experiments support this hypothesis. However, we could not identify the specific amino acid residues responsible for inhibitory potency.

In this study, we investigated the functions of CTLA-2*α* in terms of stabilizing potency under neutral pH conditions. Similar to the propeptides of cysteine protease, CTLA-2*α* exhibited a significant stabilizing activity towards its cognate enzyme, CtsL. The stabilizing activities observed in the two CTLA-2*α* mutants (C75S and W3A) were different. Both these activities were high in the C75S mutant, which suggests that the cysteine residue (C75) of CTLA-2*α* was not engaged in such functions. On the other hand, the W3A mutant lost its original activities. In a previous study, we showed that the three Trp residues of CTLA-2*α* (W12, W15, and W35) were essential for its inhibitory potency because they played a major role in maintaining structural integrity through hydrophobic interactions at the intersection of the *α*1 and *α*2-helixes (Figure 9(b)) [20]. Present studies have also demonstrated that such a core domain with two helixes of CTLA-2*α* is important not only for its inhibitory potency, but also for its stabilizing function. Similar findings have been reported for the propeptides of cathepsins [31, 33, 34]. Unlike the propeptides of cysteine protease, CTLA-2*α* is a separate protein that is expressed independently. It has a signal sequence and part of it can be secreted extracellularly [17]. The functions of extracellular cysteine cathepsins have been extensively discussed [35, 36]. Cysteine cathepsins were shown to be stable and optimally active under slightly acidic pH conditions. When the cathepsins were outside lysosomes or cells, they were rapidly and irreversibly inactivated under neutral or slightly alkaline pH conditions. Such irreversible inactivation of enzymes may be accompanied by conformational changes. A previous study has shown that invariant chain was noncovalently bound to the extracellular cathepsin L and controlled its activity [37]. Although there is no direct evidence for the interactions between CTLA-2*α* and certain cysteine cathepsins outside lysosomes or cells, CTLA-2*α* may be able to function as a regulator of such enzymes. Further studies are necessary.

## Figures and Tables

**Table 1:** Ki values of the Crammer mutants towards BCP.

Inhibitor	Sequence	Ki (nM)
70		
Wild	EFAQRCGKKVPPN	4.73 ± 0.04
F68A	E**A**AQRCGKKVPPN	∗
Q70A/R71A	EAA**AA**CGKKVPPN	∗
C72A/G73A	EAAQR**AA**KKVPPN	nsi
C72A	EAAQR**A**GKKVPPN	1.87 ± 0.04
G73A	EAAQRC**A**KKVPPN	>1000
K74A/K75A	EAAQRCG**AA**VPPN	24.5 ± 4.95
V76A/P77A	EAAQRCGKK**AA**PN	∗
P78A/N79A	EAAQRCGKKVP**AA**	∗

nsi: no significant inhibition.

∗Not significantly different from the wild.

**Table 2:** Ki values of the BCPI mutants towards BCP.

Inhibitor	Sequence	Ki (nM)
Wild	EQQSRLGLRLPAKKT	0.11 ± 0.006 [4]
Δ2	EQQSRLGLRLPAK	0.36 ± 0.037 [4]
Δ6	EQQSRLGLR	2.40 ± 0.19 [4]
Δ10	EQQSR	nsi [4]
S75A	EQQ**A**RLGLRLPAKKT	∗
R76A	EQQS**A**LGLRLPAKKT	∗
L77A/G78A	EQQSR**AA**LRLPAKKT	59.6 ± 10.7
L77A	EQQSR**A**GLRLPAKKT	2.7 ± 1.54
G78A	EQQSRL**A**LRLPAKKT	5.58 ± 1.83
L79A/R80A	EQQSRLG**AA**LPAKKT	10.7 ± 6.82
L81A/P82A	EQQSRLGLR**AA**AKKT	∗

nsi: no significant inhibition.

∗Not significantly different from the wild.

[4]: Kurata et al., 2001.
